# Understanding the child-doctor relationship in research participation: a qualitative study

**DOI:** 10.1186/s12887-020-02243-1

**Published:** 2020-07-24

**Authors:** Malou L. Luchtenberg, Els L. M. Maeckelberghe, Louise Locock, A. A. Eduard Verhagen

**Affiliations:** 1 University of Groningen, Groningen, the Netherlands; 2 Beatrix Children’s Hospital, University Medical Center Groningen, Groningen, the Netherlands; 3 Health Services Research Unit, University of Aberdeen, Aberdeen, United Kingdom; 4 Health Experiences Research Group, University of Oxford, Oxford, United Kingdom

**Keywords:** Children, Medical research, Trust, Familiarity, Doctor patient relation

## Abstract

**Background:**

Children have reported that one reason for participating in research is to help their doctor. This is potentially harmful if associated with coercive consent but might be beneficial for recruitment. We aimed to explore children’s perceptions of the child-doctor relationship in research.

**Methods:**

This is a multicenter qualitative study with semi structured interviews performed between 2010 and 2011 (United Kingdom) and 2017–2019 (the Netherlands). Interviews took place nationwide at children’s homes. We performed a secondary analysis of the two datasets, combining an amplified analysis aimed to enlarge our dataset, and a supplementary analysis, which is a more in-depth investigation of emergent themes that were not fully addressed in the original studies. All participants had been involved in decisions about research participation, either as healthy volunteers, or as patients. Recruitment was aimed for a purposive maximum variation sample, and continued until data saturation occurred. We have studied how children perceived the child-doctor relationship in research. Interviews were audiotaped or videotaped, transcribed verbatim, and thematically analyzed using Atlas.ti software.

**Results:**

In total, 52 children were recruited aged 9 to 18, 29 in the United Kingdom and 23 in the Netherlands. Children’s decision-making depended strongly on support by research professionals, both in giving consent and during participation. Often, their treating physician was involved in the research process. Familiarity and trust were important and related to the extent to which children thought doctors understood their situation, were medically competent, showed support and care, and gave priority to the individual child’s safety. A trusting relationship led to a feeling of mutuality and enhanced children’s confidence. This resulted in improving their experiences throughout the entire research process. None of the participants reported that they felt compelled to participate in the research.

**Conclusions:**

The child-doctor relationship in pediatric research should be characterized by familiarity and trust. This does not compromise children’s voluntary decision but enhances children’s confidence and might result in a feeling of mutuality. By addressing the participation of children as an iterative process during which treatment and research go hand in hand, the recruitment and participation of children in research can be improved.

## Background

Decision-making in pediatric research is difficult because children are not considered capable of giving informed consent themselves [1–3]. They do, however, have the right to participate in all decisions that concern them [4]. Therefore, assent is required as a way of affirmative agreement. Children differ in their ability and willingness to be involved in decision-making, and this is not solely age-related. [5–8]. The extent to which parents involve their child in decision-making depends on several factors including age, risk involved in the research, and whether parents see involving their child as a parental responsibility [9–12]. Children often trust their parents to protect, inform, and involve them in decision-making about participating in research, and also have confidence in healthcare professionals to help them decide [13]. This guidance of parents and doctors is mostly perceived as supportive, yet some young people have experienced a feeling of pressure from parents, doctors, or both, to take part in research [14–16].

In a previous study, we found that young people from the United Kingdom (UK) participated in clinical trials for a wide range of reasons relating to personal benefit and helping others including their doctors. We introduced this as a ‘network of exchange’ [17]. Helping a doctor as reason to participate could be harmful if associated with coercive consent. Relevant guidelines, such as the Declaration of Helsinki, [2] focus either on the process of obtaining informed consent by an independent professional, or on emphasizing the voluntary nature of participation [18]. Nevertheless, for voluntary consent and understanding of the information, children remain at least partly dependent on the clinicians’ knowledge and expertise [18].

More focus on the researcher-child interaction in the decision-making process has been found to be likely to improve children’s perception of research and to make it more meaningful [19]. Yet little empirical data is available on how the relationship between children and doctors influences children in research [20]. We question whether the child’s wish to help the doctor by taking part in research could positively influence children’s recruitment and their experience of participation without them feeling obliged to participate. Therefore, we reanalyzed data from our previous study in the UK [17] and expanded the dataset with newly obtained data from the Netherlands to further explore the role of the child-doctor relationship in pediatric research participation. This will be used to further develop our theory of the ‘network of exchange’ in pediatric research.

## Methods

### Study Design, Setting, and Participants

This is a multicenter study combining two qualitative datasets on young people’s experiences in research from the United Kingdom (UK) and the Netherlands. The UK data were used in a previous analysis [17]. The research teams consisted of two social science researchers (Louise Locock and Lesley Powell) from the Health Experiences Research Group (HERG) at the University of Oxford, and a pediatrician (Eduard Verhagen), ethicist (Els Maeckelberghe), and MD (Malou Luchtenberg) all based at University of Groningen, Beatrix Children’s Hospital, University Medical Center Groningen (UMCG), the Netherlands.

The UK study was approved by the Berkshire Research Ethics Committee (REC) (09/H0505/66). The UMCG’s Medical REC concluded that the Dutch study does not fall within the scope of the Medical Research Involving Human Subjects Act (M16.192386 2016, May 10th ), based on national criteria [21].

Participants were approached through national networks, social media advertising, word of mouth, and healthcare professionals such as pediatricians. Both studies, from which the datasets are derived, aimed for a purposive maximum variation sample (designed to reflect the broadest possible range of characteristics and experiences) [22]. in recruitment based on age (UK dataset: 10–25, Dutch dataset: 9–18 years), sex, disease or health condition, types of research they were invited to, and whether or not they had decided to participate in a study. These types of research ranged from randomized controlled trials (studying new drugs, other interventions, or ways of providing patient information), to birth cohort studies. Children could participate if they were able to express themselves in English (UK study) or Dutch (Dutch study). They received information leaflets for them and their parents separately. Children from an advisory board had been involved in developing the information. In both studies, oral and written informed consent was obtained from a parent and assent from the child. Young people over the age of 16 signed consent themselves.

### Data collection and analysis

We have taken a constructivist grounded theory approach. In this approach, introduced by Kathy Charmaz, reality is viewed to be interpretative and relative to social situations and interactions of people involved, including participants and researchers [23–25]. A grounded theory begins with a particular finding and works towards generating a theory. It is characterized by an iterative process of data collection and analysis to elucidate a process [26]. Our starting point was the finding in our previous study that young people expressed a wish to help or give back to their doctors by participating in medical research [17]. We went back to the UK data and performed a supplementary analysis, which is a more in-depth investigation of emergent themes that were not fully addressed in the original study [27]. Because we wanted to explore children’s experiences from a broader perspective as compared to our previous study, we combined the two datasets in an amplified analysis aimed to enlarge our dataset, rather than comparing the two [27].

Both datasets comprised semi structured in depth interviews (30 to 100 min) with children about their experiences with medical research at their home. Primary data from the UK study were collected by researchers from HERG from January 2010 to December 2011, and were used to contribute to the Health Talk website [28]. This is a website about real-life experiences of health and illness. The Dutch data were collected consistent with the UK methodology from May 2017 to January 2019. The interviewers, introduced to participants as researchers (LP: PhD, female, UK children, and ML: MD, PhD candidate, female, Dutch data), were trained at HERG. The interviewers had no prior relationship with the participants, and clarified to them not having influence on their medical care. A topic guide, based on the primary UK study [17] was created to structure the Dutch interviews. The main topics included reasons for (not) participating, information and decision-making including feeling obliged to take part to help their doctor, experiences during participation, involvement of doctors and parents, and children’s advice on how to improve their experience. The topic guide is presented in Table 1. Field notes were taken after the interview took place, and the topic guide was updated during data collection, which aimed at data saturation. All interviews were audiotaped or videotaped, and transcribed verbatim. Names of persons and locations were removed from the transcript. Transcripts were checked by the interviewer, and returned to the participant to check and remove any sections they wished not to be used. In the primary UK study, a few sections were removed for the website. These were not included in this analysis. The Dutch participants did not remove any sections. We only used the transcripts of both datasets for our analysis.

**Table 1:** Topic guide

*Main topics*	*Subtopics for further prompting*
**Introduction to research**	Importance of research By who approached Circumstances Healthy volunteer vs. patient participant
**Information**	Oral and written information Other sources of information Discussing information with others
**Reasons for (not) participating**	Personal benefit Helping others Other reasons
**Decision-making**	Who gave consent Influence of others such as parents and doctor Voluntary choice
**Being in a study**	Role of health professionals, family, friends Burden and risks Benefits Thinking about withdrawal Intertwinement with disease Results Acknowledgement, reward Impact on self and others Maturity Vulnerability Future participation
**Advices**	Health professionals Children Most important things discussed

Our analysis started while the Dutch data were being collected. ML and EM took on the initial coding of the interviews to find how children described the relationship with their doctor in different stages of the research process including how their doctor’s actions may or may not result in a wish to “give something back” to their doctor. After the initial coding, ML performed a more focused coding in which emergent themes about the child-doctor relationship including the roles children assigned themselves and doctors were analyzed. This was an iterative process in which themes were constantly compared to each other to find associations between and within the themes. This was supervised by EM (UK and Dutch data) and LL (UK data). The findings were discussed regularly with the entire research team through face-to-face meetings and by video-discussions. Differences were resolved through consensus. Atlas.ti software was used to help organize and analyze anticipated and emergent themes across the two datasets.

## Results

### Sample

In total, 52 participants aged 9–18 years were included in this study; 29 from the UK, and 23 from the Netherlands. Three participants from the original UK dataset had been excluded because they were over 18 to prevent adult interpretation of childhood experiences. For the primary UK study, sometimes a parent was present at the interview and sat next to his or her child. Sections where parents had expressed their views were not included in the analysis. In the Dutch study, only in one case a mother sat next to her child during the interview. She answered some questions when her child had difficulties to express himself due to his medical condition. These answers were only included when the mother clarified what her child tried to say. Participant characteristics are shown in Table 2.

**Table 2:** Participant characteristics

Characteristics	UK data N^a^ (%)	Dutch data N^a^ (%)	N^a^ (%)^b^
*Sex*			
Girl	18 (62)	9 (39)	27 (52)
Boy	11 (38)	14 (61)	25 (48)
*Age*			
9,10 years	1 (3)	4 (17)	5 (7)
11, 12 years	8 (28)	6 (26)	14 (27)
13, 14 years	8 (28)	9 (39)	17 (33)
15, 16 years	4 (14)	3 (13)	7 (13)
17, 18 years	8 (28)	1 (4)	9 (17)
*Disease*			
Acute diseases (Acute Lymphoblastic Leukemia, brain tumor, Hodgkin’s lymphoma)	4 (14)	3 (13)	7 (13)
Common chronic diseases (DM1, Crohn’s disease, asthma, arthritis, migraines)	13 (45)	7 (30)	20 (38)
Rare chronic diseases (peanut allergy, glycogen storage disease, hypophospatic rickets, vasculitis, Lupus, Wegener’s granulomatosis, osteogenesis imperfecta, cystic fibrosis, Grave’s disease, overlap connective tissue disease, tuberous sclerosis complex)	7 (24)	8 (35)	15 (29)
Other health conditions/syndromes (congenital heart condition, Prader-Willi Syndrome, restricted growth)	1 (3)	3 (13)	4 (8)
*Healthy*	4 (14)	2 (7)	6 (12)
Participants decided to:			
Participate	27 (93)	19 (83)	46 (90)
Not participate	1 (3)	3 (13)	4 (8)
Withdraw	1 (3)	1 (4)	2 (4)

^a^ Number of participants; UK data * N*= 29, NL data *N* = 23, total *N* = 52

^b^ Rounded to nearest whole number

### Children’s experiences of their relationship with health professionals in research

We found that health professionals, mostly doctors, play an important role in children’s experiences with participation in research, although our participants were not always sure what the exact roles were. In some cases, the treating physician helped only with recruitment, while in other cases, this physician appeared to be one of the doctors from the research team.

Familiarity and trust were reported to be important and related to the extent to which children thought doctors understood their situation, were medically competent, showed support and care, and gave priority to the individual child’s safety. The relationship sometimes ensued in a feeling of mutuality, meaning that they wanted to help others or give something back to the doctor specifically, and it enhanced children’s confidence, which resulted in improvement of their experiences. Children wanted to be acknowledged and they wanted to show that they saw themselves not just as patients or participants but also as persons who reciprocate. This provides a deeper understanding of the exchange between children and health professionals as part of the ‘network of exchange’ which we introduced in our previous study [17]. Table 3 provides the codes and themes which were extracted and which informed our theory.

**Table 3:** The child-doctor relationship in research as a network of exchange

*Theory*	Network of exchange							
*Themes*	Familiarity		Trust		Mutuality		Confidence	
*Roles*	**Professional**				**Child**			
	Treating physician	Researcher		Research team	Person	Patient		Participant
*Codes*	Having competence Understanding situation Giving support and care Putting child’s individual safety first Showing confidence in child Acknowledge child Being reliable				Being (more) approachable Feeling comfortable Gaining confidence Wanting to help others (voluntarily) Being (more) open to future research			

Trust, familiarity, confidence and mutuality ran through all different stages in the research process, which we will present in turn: when children were invited and considered research participation; when they made the decision whether to participate, and during their participation including looking ahead to future participation.

#### Invitation and consideration of research

The way children referred to the person who first approached them revealed that familiarity and trust were important. When invited to participate in research by their own treating physician, children described feeling more open to considering the research because they felt at ease and confident because this doctor was acquainted with their situation.

*“Well, I was in touch with my doctor for a couple of weeks before diagnosis. So yes, I did see [the doctor] as a confidant, someone who knows what he was talking about. So, when he asked me that question, it didn’t feel strange at all to me. I actually experienced it as pleasant. If two strangers stood at my bed side, asking if I wanted to participate in research, that would maybe, um, be a little bit more confusing. So, I thought…. Um, it was kind of nice that he asked me.”* (Child01; age 17, took part in RCT for Crohn’s disease treatment).

One girl said it helped that her doctor introduced the researcher who invited her to take part. Others reported that a familiar face of someone they had only noticed by accident in the hospital helped. They worried more when invited by a stranger.

*“Just talking to people I didn’t really know. And it was just a bit weird that they knew a lot about me, and I didn’t know anything about them. Because I didn’t even know any of their names. …Yes, it, like it, the second time I went they introduced themselves, but not the first time. So, I was a bit shy.”* (Child02; age 12, took part in RCT for a new drug for arthritis).

Having compassionate people around them was supportive. One participant with a chronic disease explained how she believed her longstanding relationship resulted in some sort of mutuality. She thought the relationship helped the health care professionals to feel confident about inviting her to take part, and it helped her in feeling confident to ask questions about the research.

*“I think they feel that they were more able to come up to me because they can, I’m more approachable. Because they know me very well and I’ve been there a long time and they know that I’ll help anything that they need to do. But I think they, I think they still would ask anyone, even if they were just new diabetics. I don’t think that really had much of an impact. But they were just able to talk to me more about it. …I felt more comfortable saying like, I could say what I think in front of them and they won’t take anything the wrong way. Or if I need to ask questions, no matter how stupid it is, they’ll just answer it.”* (Child03; age 17, took part in RCT comparing different ways of providing information to Type 1 diabetes patients).

Familiarity was important when children considered participation. They often discussed their wish to participate or not with their treating physician, even if he or she did not have a formal role as researcher. Children felt reassured knowing that the doctor would always put their individual safety first. They emphasized that for example in randomization, their doctor could always take them off the study if their safety was at risk.

*“Not really [worried], because like I’m sure they could find out if they really needed to, if it was an emergency. …Yes [it helped knowing them], because like they’re really nice and friendly and they tell you what they think and what they think is best, and like they only do what they think is best.”* (Child04; age 13, took part in RCT to test a new drug for arthritis).

#### Decision about taking part

Trust was expressed by some participants as a precondition for taking part.

*"…I think if they didn’t, I don’t think, I think if I didn’t trust them or if I felt that I couldn’t trust them with whatever I said, I don’t think I would have taken part. Because to me, I, trust is a main thing. And if I don’t trust someone then it’s just like, “Well, I can’t really say nothing to you.”* (Child05; age 15, took part in RCT comparing two different types of insulin for Type 1 diabetes patients).

Whereas a trusting relationship or familiarity with the research team were reportedly important to children considering research participation, this alone was not necessarily a reason for them to decide to take part. Other considerations, such as the type of intervention and burden involved also influenced their decision. Some participants reported some influence of their doctor in their decision, but that they had never felt compelled.

*“No. No no no, not at all. [laughs] No, it is just a question to which you can say yes or no and eh, if I say no then it’s not like there’s going to be a letter saying like ‘please’, you know? No, not at all. No, but I also think that if you’d feel obliged then they haven’t told you well, because it shouldn’t be the way you treat your test persons I believe.”* (Child06; age 16, took part in several trials including different diagnostic tests for patients with congenital heart disease).

One participant stated that her doctor trusting her and showing confidence in her decision helped her to make up her mind.

*“Where I did really well was that my consultant realized that I was old enough and mature enough to make my own decisions and he didn’t talk to my parents, he talked to me and that is what made me have the confidence to make that decision myself because he knew that I was mature enough to understand it, he spoke to me like I was an adult and that made all the difference.”* (Child07; age 12, declined to take part in an RCT about a new drug for leukemia patients).

Many participants described how they wanted to help their doctor by participating. They identified themselves as reciprocal persons who wanted to give something to their doctor in return. This motivation was often combined with the desire to help future sick children, or to help the hospital or research in general.

#### During and at the end of participation

Support and care from the research team and having knowledge of the situation were decisive for children in feeling reassured and confident to continue with the study. Children said that they appreciated getting to know the researcher in situations where no familiarity was previously established. This happened especially with healthy volunteers who were either invited by a stranger, or via a letter or e-mail. One of the healthy volunteers emphasized that it was very important that the people she met during the research intervention were supportive, able to give her information, and answer her questions.

*“Um, perhaps in a way I would have found it pleasant to have met the researcher, but I don’t think it’s necessary to have spoken to that person, because the other person who helped me [a research assistant] knew some things about it. So, I think it’s important that the one that is helping you knows about it [the research].”* (Child08; age 16, took part in clinical trial testing different lung function tests as healthy volunteer).

Patient participants accentuated that supportive, knowledgeable, and familiar professionals contributed to feeling comfortable and safe about staying in the research. One girl mentioned that for her a lack of trust in the research team would be a reason to withdraw from a study.

*“If I wanted to stop, my reasons would have to be I didn’t trust the people who I, like I was with, I didn’t like how I was being told to do things or being treated, and, and I didn’t feel comfortable doing the trial. All those things, I would not do the trial. Because I feel like I need to trust them, I need to know what I’m doing, and if I didn’t feel comfortable doing it, I just couldn’t do it, I just wouldn’t be able to do it.”* (Child05; age 15, took part in RCT comparing two different types of insulin for Type 1 diabetes patients).

For many children it was unclear whether the research they took part in had already ended or not. Some children wanted to be updated about the results. Especially when their reason for participating was to help others, they were curious about the value of their contribution.

*“Because yes, you are… you are in a process and you’re working towards something, kind of. So, you do hope to, eh, get to see or hear some results about it yes. Because, as I said, you are doing it for science and it would be nice if you kind of see like ‘this is what it brought because of you or the group you were participating in’.”* (Child01; age 17, took part in RCT for Crohn’s disease treatment).

Children expressed that their research experience had improved their understanding of their disease and it helped them to regain trust in their own bodies. It also made them feel more confident in managing their health condition.

***“****Now, we can… by now we also understand it a little. We have injected it more often now, so we know a bit about how and where we should put things [materials]. And how… which protocol we have. Yes, it’s going pretty well. Yes. Good.”* (Child09) *“Yes, she [our mother] has followed a training in that. Yes, it was part of it. That she knows how to do it, so in principal she could easily do it [the injection].”* (Child10; sibling of Child09, age 14, took part in a drug trial for hypophosphatemic rickets) *“…But we thought, we could do it ourselves as well. …Yes, I am … I like that idea. Yes. As I just said, it gives me some sort of feeling like yes… it’s some kind of confidence you know?”* (Child09; age 13, took part in a drug trial for hypophosphatemic rickets).

Some participants said they felt more open to future research opportunities because of their positive experiences, especially when it concerned research that was introduced by their own treating physician.

## Discussion

This study explored how children experienced the child-doctor relationship in research. For patients, we found that familiarity and trust in their treating physician was important for considering participation, for consenting to participate, for remaining in a study, and for considering future participation. Interestingly, the trusting relationship between patients and their treating physician sometimes led to a feeling of mutuality, and this enhanced children’s confidence. Healthy volunteers, who did not have a prior relationship with a doctor or researcher, depended more on parental support when introduced to research. Healthy volunteers also expressed that becoming familiar with the researcher was important to feeling reassured about continuing the research. None of the participants reported that they felt obliged to take part in research. We will elaborate on why trust in professionals matters, consider the intertwinement of research and care, and stress the importance of treating children’s research participation as an iterative process to improve their engagement in research.

### Trust placed in the treating physician

Our findings confirm results of Tromp and Van de Vathorst that parents and adolescents report personal trust as being an important factor in the decision to participate in clinical trials [29]. According to O’Neill, trust cannot be replaced by other values such as transparency, autonomy, or accountability [30]. She states: *“where we aim not to influence others, but to place and refuse trust intelligently, we must link trust to trustworthiness, and must focus on evidence of honesty, competence and reliability.”* [31]. Children in our study said they trusted their doctor who knew about their situation and had shown to be able to make truthful claims based on their specific situation (honesty). In addition, children experienced that their doctors had the ability to do what they said (competence), and that the doctors had provided support and care. Therefore, the doctors had shown commitment to the children (reliability). The value of trustworthiness of the doctor-researcher could explain why healthy volunteer participants, who lack such a prior relationship, were more cautious during their first contact with the research team and hospital.

### Intertwinement of research and care

Trust and familiarity seemed to be particularly important for supporting children whose treatment intersected with the research they were invited for, especially in situations where the consequences were not always foreseeable, such as when children were overwhelmed because they had recently been diagnosed, or when children were to be randomized to receive a specific drug. The importance of trust and familiarity in coping with these uncertainties in research seems to be similar to coping with disease in the (regular) health care setting. Parents of children with a rare disease for example, expressed the importance of mutual trust in decisions about treatment, [32]. and others recognized the importance of tailored care and support for their children with trisomy 13 and 18 [33]. Trust and respect in relationships with healthcare providers were found to be essential facilitators for shared decision-making in pediatrics [34]. Our participants did not always report a trusting patient-doctor relationship to be a precondition for taking part, but they did mention feeling less worried and more open towards research participation. This confirms findings from the study of Dekking et al. in which parents said they valued the involvement of their child’s physician in the informed consent process [35]. When the informed consent procedure is done by someone that is *“completely independent of this relationship”*, as required in the Declaration of Helsinki (article 27), [2] this might have the consequence that children will not be open to considering the research, even though they may otherwise be willing to participate.

In some areas, such as pediatric oncology, a strict division between the physician and researcher role is not always possible, and dividing the two roles can cause problems in communication between them [36]. This blended role of the treating physician and researcher requires responsibility and a constant awareness of the professional of potential conflicts. Given the similarities in patient-doctor relationships in health care and research, it could be questioned whether it is realistic to hold on to a strict separation of the doctor’s role as treating physician and as researcher, or that it might be better for the two roles to go hand-in-hand, as in pediatric oncology.

### Child-doctor relationship in research as iterative process

Support and interaction with the research team during participation seemed to help our participants in coping with uncertainties and decision-making. Decision-making was not restricted to the single moment of giving informed consent but was also directed at decisions about retention in a study, or decisions about future research. An alternative, more personalized approach towards informed consent, leaving more room for trustworthiness, honesty, and openness in the patient-doctor relationship, has been suggested for clinical genetic testing [37]. Budin-Ljosne et al. pointed out that more focus on the relational and societal aspects of research and ongoing communication between the participant and researchers through dynamic consent, could improve recruitment in research [38]. We recommend seeing children’s participation in pediatric research as an iterative process, whereby decision-making and interaction between the research team and the participant takes place during the entire process, and is not only related to consent. This approach seems to connect well to the positive experiences the young people mentioned at the end of their participation, which could encourage trial participation in the future. Recently, Sisk and Baker published a model for trust and relationship maintenance in pediatric care [39]. They hypothesize that doctors should demonstrate caring, fidelity, and honesty, in addition to competence, to maintain and build trust over time. Our findings illustrate that this iterative model might also work for research relationships.

### Strengths, limitations, and future research

By combining the UK and Dutch datasets we obtained extensive data about children’s experiences with participation in different kinds of medical research. The children included both patient participants and healthy volunteers, and children who declined or withdrew from research. Having obtained more data from children who did participate in research could have had a positive influence on our view of the trusting relationship between children and professionals. Nevertheless, the data obtained from children who decided not to participate also showed that trust was important. Those children expressed other reasons to decide not to take part in research, and not a lack of trust.

In some of the interviews in the UK, a parent sat next to the child when the interview was taken. Unfortunately, it was not formally recorded how often this was the case, and we only had access to the interview transcripts for this secondary analysis. Although the views expressed by parents were not taken into account and children themselves seemed very open about their experiences, children may have been influenced in what they said as a result of the presence of a parent. This is a potential limitation of our study. Another limitation might be that some sections in the primary UK study were permanently removed at the request of participants before being used on the website, and could therefore not be taken into account for this secondary analysis. The removed sections, however, occurred in only five transcripts, and contained a few seconds of data to a maximum of one minute. Although the sections around those parts of the data mainly concerned other topics, we cannot fully be sure that it has not affected the validity of our results.

The influence of familiarity and trust on children’s decision-making might be affected by the child’s age and the type of research the child in invited to. In our population, age did not seem to be influential, but the type of the study and whether it affected children’s disease management did seem to matter. Children who took part in, for example, a randomized controlled trial that involved a change in their treatment put more focus on the importance of trust as a precondition for taking part, and also during the research itself, whether healthy volunteers who took part in a trial that did not directly affect their health felt that becoming familiar with the researcher was important but not a prerequisite. Future research should focus on the influence of those aspects. We believe this needs future studies with a more quantitative or mixed methods approach.

## Conclusions

Children’s experiences suggest that in pediatric research the child-doctor relationship should be characterized by familiarity and trust. When research intersects with children’s treatment, the combined physician-researcher role did not seem to be associated with involuntary participation, but rather with desire of mutuality, and it enhanced children’s confidence. This shows the importance of building a trustful relationship with children in research that goes beyond procedural requirements. Addressing children’s participation as an iterative process in which treatment and research go hand-in-hand could potentially improve recruitment and engagement of children in medical research.

## Data Availability

The qualitative data that were analyzed and presented in this study are not publicly available on account of the privacy of the participants.

## Abbreviations

HERG: Health Experiences Research Group
REC: Research Ethics Committee
UK: United Kingdom
UMCG: University Medical Center Groningen
